# Primary care providers’ perspectives on the implementation of couple-based collaborative management for Chinese older adults with type 2 diabetes: a mixed-methods study

**DOI:** 10.3389/fpubh.2026.1781781

**Published:** 2026-04-10

**Authors:** Yingxin Xu, Conghui Yang, Jingyi Zhi, Yujie Ma, Huiqiong Zheng, Qiao Liu, Jing Liao

**Affiliations:** Department of Medical Statistics and Epidemiology, Sun Yat-sen University, Guangzhou, China

**Keywords:** couple-based collaborative management, implementation science, normalization process theory, primary care, type 2 diabetes

## Abstract

**Background:**

Couple-based collaborative management (CCMM) has shown promise in supporting chronic disease self-management among older adults and addressing the growing public health burden, but its integration into primary care remains uncertain. This study explored primary care providers’ (PCPs) perceptions of CCMM using normalization process theory (NPT) to identify key factors influencing its routine implementation.

**Methods:**

Thirty five PCPs involved in a community-based randomized controlled trial completed the Normalization Measure Development (NoMAD) questionnaire. Descriptive statistics and Cronbach’s alpha were used to assess quantitative data. Semi-structured interviews were conducted with five purposively selected PCPs and were analyzed thematically according to NPT constructs.

**Results:**

PCPs reported high familiarity with CCMM (mean = 8.03, SD = 1.36) and positive views regarding its potential integration into practice. Mean scores for the four NPT constructs ranged from 1.76 to 2.47, and internal consistency of the NoMAD was strong (Cronbach’s alpha = 0.94). PCPs recognized CCMM as distinct from usual practice and valued its relevance (coherence), which encouraged engagement (cognitive participation). However, barriers included heavy workloads, staff shortages, limited confidence in some clinical tasks, challenges in sustaining continuity, and concerns about patient adherence and couple relationships. These factors hindered collective action and reflexive monitoring.

**Conclusion:**

PCPs understood and supported CCMM conceptually and expressed willingness to adopt it, yet structural and organizational barriers limited its practical implementation. Implementing CCMM in primary care could improve chronic disease management and promote healthy aging. Addressing workload pressures, enhancing provider training, and ensuring continuity will be essential to normalize CCMM in primary care.

## 1 Introduction

Rapid population aging (1) poses a major challenge to achieving healthy aging (2). In this context, China needs to strengthen primary health care to improve access to care and quality of services for older adults (3). However, primary health care is constrained by a shortage of qualified primary care providers (PCPs) and high workloads (4), which have been further exacerbated by the COVID-19 outbreak (5). Family involvement, which can compensate for limited doctor-patient interactions, has been identified as a key factor in enhancing self-management and health outcomes in chronic disease care (6). Rolland’s Family Systems Illness model views chronic illness as a shared family experience that evolves over time, with families adapting to the illness through mutual support, collaborative problem-solving, and role adjustments (7). International studies have shown that family involvement can improve patients’ clinical outcomes, health behaviors, and psychosocial well-being, while also enhancing marital relationships and quality of life (8–10). Similar benefits have been observed in China, including improved self-efficacy and lifestyle adherence (11), reduced psychological distress among couples (12), and better glycemic control (13).

Based on the Dyadic Model of Coping with Chronic Illness (DMCCI) (14) and Social Cognitive Theory (SCT) (15), couple-based collaborative management (CCMM) has been proposed as a new strategy and has been implemented (13). This strategy posits that when couples perceive illness as a shared challenge rather than an individual burden, their collective efficacy, defined as their mutual belief in working together to manage the illness, will increase. Enhanced collective efficacy can foster collective behavior changes, positively impacting both the patient’s clinical outcomes and the couple’s quality of life (13, 16).

Although the theory of CCMM is well-developed, its implementation in routine primary care remains limited. Systematic reviews have highlighted a significant gap between the efficacy of couple-oriented interventions demonstrated in research settings and their widespread adoption in routine practice (17). Previous studies have mainly examined the acceptability and effectiveness of CCMM from the perspective of participants, indicating that couples generally respond positively and integrate these interventions well into daily life (18–20). Nevertheless, few studies have explored the feasibility of implementing CCMM in primary care from the perspective of providers. Evidence suggests that providers are generally supportive of CCMM in principle (21, 22). However, evidence regarding its practical implementation is scarce. Only one study, which focused on end-of-life family support in an acute hospital setting, showed that providers could co-deliver, reflect on, and evaluate the intervention, and that it could be integrated into their daily routines (23). Thus, it remains unknown whether CCMM can be integrated into the chronic disease management practices of providers in the primary care system.

Our team previously conducted a randomized controlled trial (RCT) (16) to evaluate the effectiveness of CCMM in promoting the health of older Chinese patients with type 2 diabetes and their spouses. Based on the RCT, this study intended to further investigate whether CCMM can be integrated into primary chronic disease management. By shifting the focus from effectiveness to implementation, this study sought to bridge the gap between controlled trial conditions and primary care implementation. Guided by the normalization process theory (NPT), an implementation framework that examines the work required to introduce and sustain new practices in healthcare settings (24), the aim of this study was to use mixed methods to assess PCPs’ perceptions of CCMM and to identify the key factors for influencing its integration into primary care routines. By highlighting the provider perspective, this study provided evidence of the application of CCMM in practice, demonstrating how to implement CCMM routinely in the primary care system. Understanding these implementation factors is crucial for developing evidence-based recommendations that can inform public health policy and practice, ultimately contributing to more sustainable and effective chronic disease management for the aging population.

## 2 Methods

### 2.1 Study design

This study was conducted as part of a multicenter randomized controlled trial in our city, spanning the period from September 2020 to March 2021, during the COVID-19 outbreak in China, when primary care services were substantially influenced by epidemic prevention and control measures (16). The intervention was based on the couple-based collaborative type 2 diabetes management program. A total of 207 patients with type 2 diabetes and their spouses were recruited from 14 community healthcare centers. After completing baseline data collection, participants were randomized within each center to the couple-based intervention group or the individual-based control group. PCPs were responsible for recruiting participants and delivering weekly group education and training sessions over a four-week period. The intervention group sessions involved both patients and their spouses and focused on collaborative management, while the control group sessions involved patients only and focused on individual self-management. Follow-up assessments were conducted at 3, 6, and 12 months after baseline.

To assess key factors in the implementation of complex interventions and improve the quality of follow-up interventions, we used a mixed-methods design based on NPT. We collected and interpreted data from both quantitative and qualitative methods. In the quantitative study, the Normalization Measure Development questionnaire (NoMAD) survey was administered to PCPs participating in the implementation after the intervention. Semi-structured interviews with PCPs, conducted either face-to-face or via teleconference after the end of the program follow-up, served as the qualitative component. The study was approved by the Sun Yat-sen University Institutional Review Board (Approval no. 2019–064).

### 2.2 Study participants

All PCPs participating in the intervention were invited to take the NoMAD questionnaire survey. All 35 participating PCPs were included in this study, representing the complete population of implementers rather than a sample subset. Subsequently, semi-structured interviews were conducted with a purposively selected subset of five PCPs from two communities with high overall attendance rates and three with low overall attendance. The sample size for the qualitative interviews was determined by the principle of data saturation. Saturation was considered to have been reached after the planned interviews were completed, as the content collected was rich and no new information emerged in the final few interviews.

### 2.3 Quantitative survey data

The NoMAD instrument was developed based on the structure of NPT (25, 26) and has demonstrated good face validity, construct validity, and internal consistency (26, 46). It can be used to assess PCPs’ perceptions of embedding the intervention in their daily work (27, 28). The NoMAD instrument is a 23-item questionnaire consisting of three general items about the intervention and 20 items based on the NPT structure (coherence-4 items, cognitive participation-4 items, collective action-7 items, and reflexive monitoring-5 items). Item 2 in the collective action scale is reverse-scored, while the rest are positively scored. The NPT items employ a 5-point Likert scale, ranging from 1 (strongly agree) to 5 (strongly disagree). A set of ‘not relevant’ response options is also provided to capture the reasons for not being able to respond on the 5-point Likert scale (26). The three general items are scored using a response scale of 0–10, where 0 = not at all, 5 = somewhat, and 10 = completely (29). In addition, we added two questions for evaluating PCPs’ preferences for the self-management model and CCMM, as well as their willingness to implement CCMM over the self-management model at work.

Given the limited number of PCPs, quantitative analyses were descriptive and exploratory. Descriptive analysis was performed on the NoMAD instrument using means, standard deviation (SD), 95% confidence intervals, absolute frequencies, and relative frequencies. Items rated as ‘not relevant’ were not included from the analysis. Lower mean scores on items indicate higher agreement. The reliability of the NoMAD questionnaire was evaluated using Cronbach’s alpha (30). A Cronbach’s alpha between 0.70 and 0.95 was considered indicative of good reliability. Analyses were completed using R 4.4.2.

### 2.4 Qualitative interview

To identify the key factors contributing to successful implementation and to develop recommendations for enhancing implementation and optimizing the integration of interventions in daily clinical practice, two investigators conducted semi-structured interviews with PCPs. The interview themes were predetermined based on the four structures of NPT (coherence, cognitive participation, collective action, and reflexive monitoring) (31), including knowledge of the program, specific implementation, difficulties in implementation, perceived effectiveness, follow-up suggestions, and understanding of CCMM. These themes were incorporated into a semi-structured interview guide, which was used by the two researchers to conduct the interviews. The interviews lasted 30–40 min and were recorded and transcribed.

Interview data were analyzed using thematic analysis and coded using Nvivo11. An inductive approach to analysis was employed to capture the themes that participants deemed important, ensuring that the data were not constrained by the predefined NPT structure. This was followed by a deductive phase, where the identified themes were mapped onto the four NPT structures to provide a structured understanding of the implementation process. Two researchers independently coded the data to generate the initial themes and then reviewed the themes. When differences arose, the research team discussed to achieve consensus.

## 3 Results

A total of 35 PCPs in 14 communities participated in the intervention, and all completed the NoMAD survey. Table 1 showed the basic information of the PCPs. The mean age of PCPs was 35.5, 34.3% were male, 60.0% were team leaders or deputy team leaders, 57.1% were clinicians, 77.1% were located in urban areas, and 51.4% had more than 6 years of work experience. The five PCPs who were invited to participate in the interviews included two clinicians, two nurses, and one public health physician.

**Table 1:** Primary care providers’ characteristics (*n* = 35).

Characteristics	*n* (%) or *M* (SD)
Male, *n* (%)	12 (34.3)
Age *M* (SD)	35.5 (6.6)
Role, *n* (%)	
Team leader	11 (31.4)
Deputy team leader	10 (28.6)
Others	14 (40.0)
Occupation type, *n* (%)	
Public health physician	5 (14.3)
Clinician	20 (57.1)
Nurse	7 (20.0)
Others	3 (8.6)
Work experience, *n* (%)	
Less than 1 year	4 (11.4)
1–2 years	7 (20.0)
3–5 years	6 (17.1)
6–10 years	6 (17.1)
11–15 years	8 (22.9)
More than 15 years	4 (11.4)
Community District, *n* (%)	
City	27 (77.1)
Rural	8 (22.9)

### 3.1 PCPs’ perceptions

**Figure 1:**
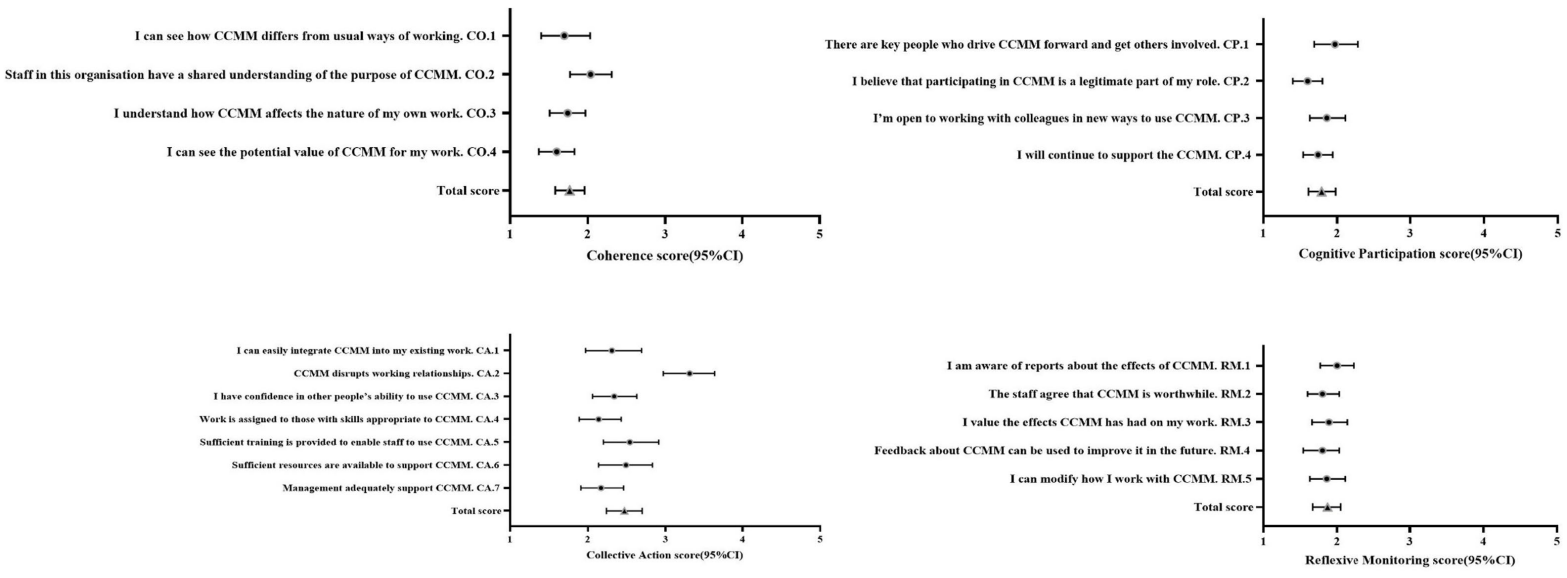
Normalization measure development (NoMAD) scores by item. CCMM: couple-based collaborative management model, CO: coherence, CP: cognitive participation, CA: collective action, RM: reflexive monitoring, CI: confidence intervals.

For the NoMAD general questions about the intervention, PCPs reported a mean score of 8.03 (SD = 1.36) regarding how familiar they were with CCMM. PCPs reported mean scores of 7.71 (SD = 1.79) and 7.29 (SD = 2.27) when asked whether CCMM was currently a normal part of their work and whether they felt that CCMM could become a normal part of their work. Additionally, PCPs reported mean score of 8.51 (SD = 1.44) and 7.86 (SD = 1.70) when asked whether they perceived CCMM to be superior to the self-management model and whether they would be more willing to implement CCMM in their work. The mean scores (SD) for the four constructs in the NoMAD study were 1.76 (SD = 0.59), 1.79 (SD = 0.58), 2.47 (SD = 0.70), and 1.87 (SD = 0.58), respectively. These findings suggested that PCPs generally expressed positive perceptions of CCMM, although agreement was comparatively weaker for collective action. Figure 1 showed the scale scores. In terms of coherence, 94.4% of PCPs agreed that “CCMM differed from usual ways of working.” In relation to cognitive participation, 94.4% of PCPs indicated that “Participating in CCMM was a legitimate part of my role.” In terms of collective action, 69.4% of PCPs agreed that “Work was assigned to staff with skills appropriate to CCMM.” With regard to reflexive monitoring, 88.9% of PCPs agreed that “Staff agreed that CCMM was worthwhile.” Cronbach’s alpha values of the four constructs of the NoMAD questionnaire (coherence, cognitive participation, collective action, reflexive monitoring) were 0.75, 0.82, 0.86, and 0.90, respectively. The overall Cronbach’s alpha value for the NoMAD questionnaire was calculated as 0.94. All values fell between 0.75 and 0.95, indicating good internal consistency.

### 3.2 Key factors for CCMM

Table 2 presented the key factors influencing implementation according to the constructs of NPT.

**Table 2:** Key factors associated with the implementation of couple-based collaborative management model.

Construct	Interview theme	Key factors
Coherence	Understanding of the program	Understanding the specific requirements of CCMM
		Patient adherence leads to CCMM implementation
		Couple relationships affect CCMM effectiveness
Cognitive participation	Methods for implementing the intervention	Detailed CCMM presentations may discourage participants
Collective action	Difficulties of the intervention process	Additional CCMM interventions increase daily workload
		Difficulty in recruiting participants suitable for CCMM
		Insufficient intensity of CCMM intervention
		Change management is harder in classrooms alone for older adults
		Part of the intervention is challenging for nurses and public health physicians
		Insufficient resource support due to COVID-19
Reflexive monitoring	Effectiveness of the intervention	Unclear about the effect of CCMM
		Colleagues feel little change in behavior
		The effectiveness of CCMM was modest

CCMM: Couple-based Collaborative Management Model.

#### 3.2.1 Coherence

PCPs identified understanding the specific requirements of CCMM as a key factor.

*“I need to understand what I’m going to do first, and then I’m going to mobilize other people to do it. The process is difficult and it takes quite a lot of time to think about it and then implement it.” (Nurse 2).*

Other key factors noted by PCPs included patient adherence playing a dominant role and discordant couple relationships significantly influencing intervention outcomes. These were considered major determinants of CCMM implementation.

*“Some patients have a strong sense of self or self-awareness, and he can dominate his family to assist them or to cooperate with him in controlling his condition.” (Clinician 1).*

*“If implemented in a place where family doctors are the main management model, I think it is effective. But to do it in our city, I do not think it’s necessarily effective. Because it depends on three things, one is their level of awareness, two is their age, and three is the couples’ relationship.” (Nurse 2).*

#### 3.2.2 Cognitive participation

Detailed CCMM descriptions were perceived as potentially reducing participants’ motivation and hindering cognitive participation.

*“The recruitment only briefly introduced the general situation of the program, without emphasizing the specific details. If too many details are introduced, it may have some effect on their motivation.” (Clinician 1).*

#### 3.2.3 Collective action

PCPs identified the increased daily workload associated with additional CCMM intervention as a key factor and cited difficulties in recruiting participants suitable for CCMM.

*“Because this is an occasional job, it adds to the workload. Our community hospital’s original work was busy, and the addition of this work increased our burden.” (Clinician 1).*

*“I mobilized a lot of people, but after they came over for the physical examination, only half of them were eligible. So it took a lot of effort in the early part, but the results were not very good.” (Public health physician).*

Insufficient intensity of the intervention and the challenge of changing patient behavior through classroom education alone for older adults were also noted.

*“The intensity of the intervention was not sufficient, leading to poor continuity of the intervention on the one hand, and the other hand to a failure to adapt the intervention to the participant’s situation on time.” (Nurse 1).*

*“I think I have introduced the content of the course clearly, but the acceptance level may be discounted. Because the participants of our course are older adults, their perceptions of the disease are already relatively fixed, and I think it is unlikely to change their behavior through 4 lessons.” (Nurse 2).*

In addition, nurses and public health physicians reported that the clinical content of the intervention, particularly regarding medication management, posed a challenge due to their limited prior expertise in this area.

*“This lecture is technically demanding and I need to learn it. I had less knowledge about diabetes management before, and to deliver these 4 lessons, especially the one on medication, I had to go through self-input, then filter, and then output to the residents, so I think the lesson on medication was the most difficult.” (Nurse 2).*

*“The medication aspect is difficult for me, I do not know this aspect as well as the clinical, and I’m not as good as them in medication instruction.” (Public health physician).*

Furthermore, the prevalence of COVID-19 during the intervention period necessitated changes in work priorities and increase workloads, which were common barriers faced by all interviewees.

*“Our work is mainly focused on epidemic prevention and control, and intervention may be a bit limited in terms of energy, and I feel that intervention is not as good as it used to be.” (Nurse 1).*

*“Especially if there is an epidemic, we cannot do face-to-face visits anymore. Interviewing patients face-to-face is a more acceptable way, so the impact may be significant.” (Public health physician).*

#### 3.2.4 Reflexive monitoring

PCPs identified a lack of clarity about the effects of CCMM and the perception of poor outcomes as influencing reflexive monitoring, while noting that staff may perceive the intervention as having limited impact on behavior change.

*“Because I do not know what the final data is, so I do not know how well they are actually being managed.” (Public Health Physician).*

*“My colleagues felt nothing changed at first, and they expected it to be quite accurate.” (Nurse 2).*

*“I feel that for our grassroots, the implementation is not solid enough, and I feel that it has not achieved the desired purpose and what we thought management could achieve.” (Nurse 1).*

*“I think it took a lot of time that should have changed the spouse’s mind. I expected that 90% of couples should be able to achieve improved glycemic control, but now I estimate only 50%.” (Clinician 2).*

## 4 Discussion

**Figure 2:**
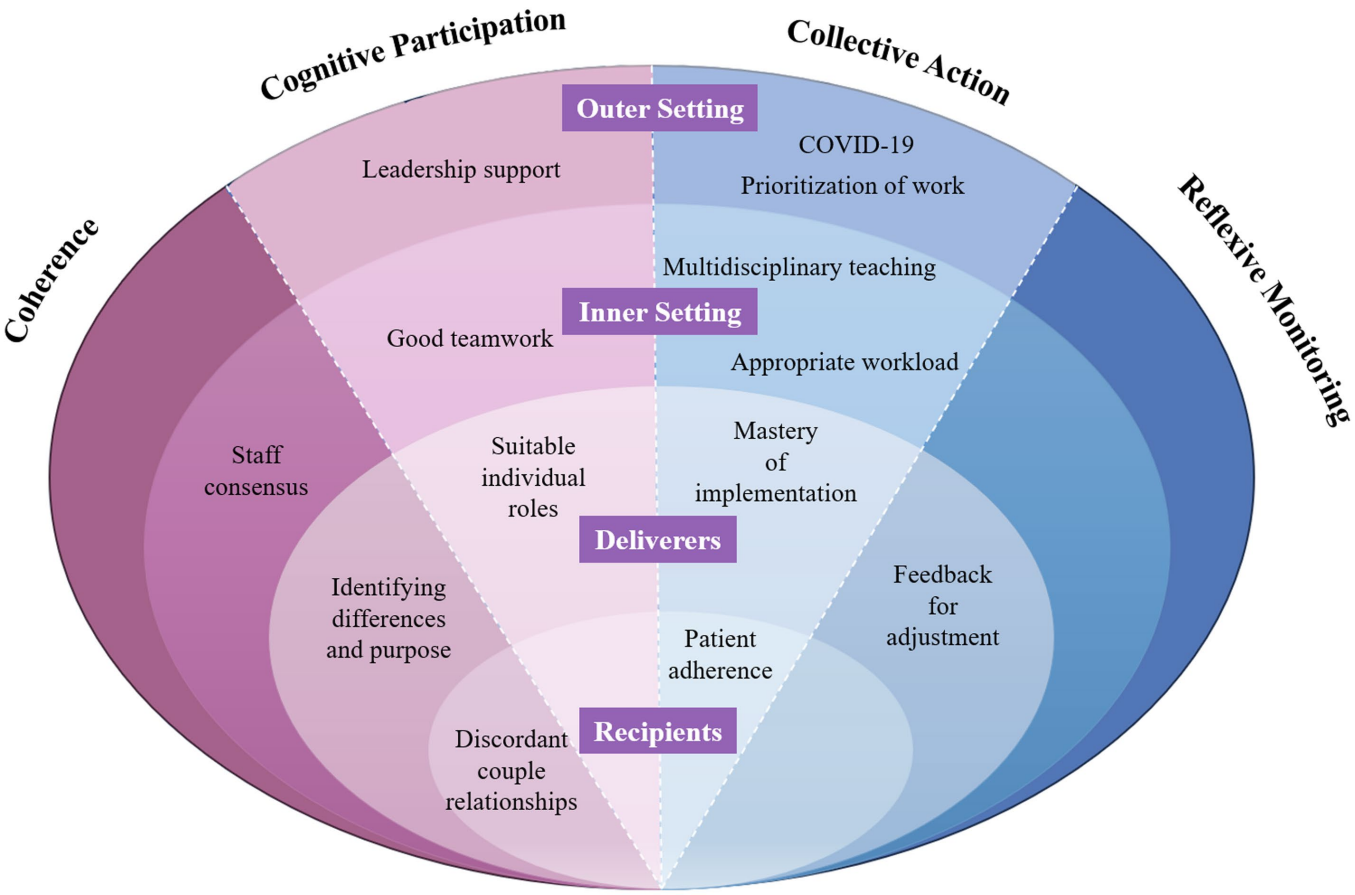
Strategies for the normalized implementation of couple-based collaborative management model in primary geriatric health management.

This study assessed PCPs’ perceptions of CCMM in the primary care system and identified the key factors influencing its integration into daily work. Our findings showed that PCPs strongly agreed that CCMM was superior to traditional self-management and were more willing to implement CCMM at work. However, the actual implementation was not as expected, especially in terms of collective action. Figure 2 showed the strategy for incorporating CCMM into the chronic disease management of PCPs in primary care based on the findings of this study.

At the cognitive level, PCPs recognized the value of CCMM (coherence), which promoted their engagement (cognitive participation). Although consistent with previous findings, PCPs were concerned about the effectiveness of CCMM for couples in troubled relationships (22). PCPs in our study were still able to fully understand the difference between CCMM and self-management and appreciate its value for diabetes management in older adults. This consensus among PCPs may be attributed to the context of Chinese traditional family culture. In this context, the involvement of family members in disease management is not only commonplace but also socially sanctioned (32, 33). As a result, it was relatively easy for PCPs to reach a consensus regarding CCMM, which embraces such familial engagement. Consistent with previous research, consensus among PCPs promoted effective collaboration within the team (34) and leadership support (35), both of which facilitated intervention initiation. Compared with previous studies, the occurrence of patient distrust leading to negative PCPs’ attitudes (36) was less common. This may have facilitated collaboration within the team, where PCPs could communicate and connect effectively with the couples involved.

However, notwithstanding the commendable level of comprehension and engagement demonstrated by PCPs, the actual execution (collective action) and subsequent refinement through feedback (reflexive monitoring) fell short of expectations. In collective action, heavy workloads and staff shortages in the primary care system resulted in insufficient time for PCPs to complete additional intervention tasks. Previous studies have also reported similar barriers (37, 38). More seriously, the COVID-19 outbreak during the implementation period shifted work priorities (39, 40) and exacerbated the already heavy workload of PCPs (41). The outbreak also disrupted the continuity of interventions, creating barriers to patient management (42). At the individual level, some public health physicians and nurses lacked confidence in medication guidance, which affected intervention delivery. This finding is consistent with previous research indicating that differences in scope of practice contribute to variations in confidence levels among physicians and other healthcare professionals when conducting such consultations (38), and that training can improve providers’ competence and confidence (43). In reflexive monitoring, PCPs indicated that they were not able to perceive the specific effects of implementation during follow-up visits, which hindered their motivation. This may be because some patients had strong autonomy, and the couples involved were older and had established fixed habits, making it difficult to change long-standing lifestyles through short-term intervention (44).

### 4.1 Clinical implications

This study provides practical insights for integrating CCMM into chronic disease management in primary care (Figure 2). For PCPs, the findings highlight the importance of identifying suitable couples and tailoring interventions to improve adherence and health outcomes. Effective implementation also requires clear role allocation within primary care teams, targeted training to strengthen PCPs’ capacity and confidence, and continuous feedback from patients and their spouses to refine management strategies. In addition, leadership support, appropriate workloads, and contingency plans are essential to sustain family-centered care in routine primary care practice.

### 4.2 Public health implications

This study demonstrates that CCMM is an approach accepted and endorsed by PCPs for improving chronic disease management among older adults. Involving spouses and other family members and empowering them with the necessary skills and knowledge can enhance patient adherence and improve self-management behaviors (8, 11). This family-centered care approach requires fewer resources than conventional health-care schemes and can be adapted to different communities and healthcare settings. Furthermore, family involvement can help PCPs reach underserved communities, improve health outcomes, and build more resilient healthcare systems (45). Scaling up such interventions in primary care can alleviate the burden of chronic diseases among aging populations. Policymakers and health administrators should consider integrating CCMM into routine primary care services as part of broader public health initiatives targeting older adults.

### 4.3 Strengths and limitations

The strength of this study lies in its use of a mixed-methods approach to analyze the implementation process, while the participating PCPs included clinicians, public health physicians and nurses with varying degrees of knowledge and experience from 14 communities in our city. However, some limitations of this study cannot be ignored. First, the NoMAD questionnaire was administered only at the end of the intervention. While this post-implementation assessment effectively captured the key factors and PCPs’ perceptions at the conclusion of the process, it did not allow measurement of attitudinal changes from baseline. Nevertheless, this approach aligned with the study aim, which focused on identifying key factors for integration. Although the sample size of quantitative survey was relatively small, its results could complement and validate qualitative findings. Second, there were different levels of participants in the program, including decision-makers, PCPs, and supervisors. Our data collection focused on PCPs who were most directly involved in the frontline delivery of the intervention. This limited our ability to full reflect the exchange of information among participants. Third, the data were collected during the COVID-19 pandemic. Some implementation barriers observed in this study may reflect the pandemic context rather than routine conditions. However, this context also provides valuable insight into the adaptability required for implementing chronic disease management under public health emergencies. Finally, the program was implemented in our city, a coastal city with a relatively strong economy, and the findings of the study may not be generalizable to rural areas.

## 5 Conclusion

This study assessed the perceptions of PCPs in the primary care system regarding CCMM and identified key factors for integrating CCMM into daily practice. It was easy for PCPs to identify with CCMM at the cognitive level. However, to ensure the actual implementation effect of CCMM, it is necessary to increase the number of PCPs, reduce workloads pressures, improve providers’ confidence and competence through training, and maintain intervention continuity. Our study proposed a strategy for the normalized implementation of CCMM in primary geriatric health management, which may help promote a family-centered approach to chronic disease management and improve population health outcomes among older adults, contributing to public health goals for healthy aging.

## Data Availability

The audio records and the transcripts collected during the current study are stored in a file on a local computer of Department of Public Health, Sun Yat-sen University for data security, which are available from the corresponding author on reasonable request. Participants’ personal information is confidential and is not shareable.
